# Is Passive Syntax Semantically Constrained? Evidence From Adult Grammaticality Judgment and Comprehension Studies

**DOI:** 10.1111/cogs.12277

**Published:** 2015-11-26

**Authors:** Ben Ambridge, Amy Bidgood, Julian M. Pine, Caroline F. Rowland, Daniel Freudenthal

**Affiliations:** ^1^ University of LiverpoolESRC International Centre for Language and Communicative Development (LuCiD)

**Keywords:** Child language acquisition, Passive, Verb, Semantics, Autonomy of syntax, Theme‐experiencer, Experiencer‐theme, Agent‐patient

## Abstract

To explain the phenomenon that certain English verbs resist passivization (e.g., *£5 was cost by the book), Pinker (1989) proposed a semantic constraint on the passive in the adult grammar: The greater the extent to which a verb denotes an action where a patient is affected or acted upon, the greater the extent to which it is compatible with the passive. However, a number of comprehension and production priming studies have cast doubt upon this claim, finding no difference between highly affecting *agent‐patient/theme‐experiencer* passives (e.g., *Wendy was kicked/frightened by Bob*) and non‐actional *experiencer theme* passives (e.g., *Wendy was heard by Bob*). The present study provides evidence that a semantic constraint is psychologically real, and is readily observed when more fine‐grained independent and dependent measures are used (i.e., participant ratings of verb semantics, graded grammaticality judgments, and reaction time in a forced‐choice picture‐matching comprehension task). We conclude that a semantic constraint on the passive must be incorporated into accounts of the adult grammar.

## 1 Introduction

Language is a defining feature of human cognition. Thus the nature of the representations that underlie adult linguistic competence constitutes a central question in the cognitive sciences. Traditional approaches (e.g., Chomsky, 1993; Newmeyer, 2003) treat at least some of these representations as purely syntactic; context‐free rules or processes that are impervious to semantic content. Rival approaches (e.g., Goldberg, 1995; Pinker, 1989) emphasize the communicative nature of language. On this account, the fundamental representations underlying linguistic competence are inherently meaningful in nature. The goal of the present article is to pit these two positions against one another by means of an empirical investigation of the representation and processing of the English passive — an archetypal example of a phenomenon that is seen as reflecting either context‐free rules under the former account or a meaningful linguistic construction under the latter.

In English, as in many languages, the same event can (in most cases) be described by either an active sentence (1–3a) or an equivalent passive (1–3b).

**Table:** 

1a. Wendy kicked Bob	1b. Bob was kicked by Wendy
2a. Wendy frightened Bob	2b. Bob was frightened by Wendy
3a. Wendy saw Bob	3b. Bob was seen by Wendy

But just how do speakers form a passive? Are the representations underlying passive formation purely (morpho‐)syntactic or subject to semantic constraints?

Arguing for the latter possibility, Pinker, Lebeaux, and Frost (1987: 249; see also Pinker, 1989) propose that passivization is restricted to verbs that denote actions or events such that

[B] (mapped onto the surface subject [of a passive]) is in a state or circumstance characterized by [A] (mapped onto the *by*‐object or an understood argument) having acted upon it.

As a shorthand, in subsequent discussion, we will refer to this constraint as the “affectedness” constraint. Pinker's primary motivation for this constraint appears to be the existence of a number of verbs for which the *by*‐object does not act upon or affect the surface subject, and which resist passivization altogether (4–5).

**Table:** 

4a. The book cost £5	→	4b. *£5 was cost by the book
5a. This tent sleeps five people	→	5b. *Five people are slept by this tent

In contrast, most current psycholinguistic approaches treat the passive as a wholly syntactic phenomenon. This is particularly true for theories within the Chomskyan framework (e.g., Boeckx, 1998; Carnie, 2007; Collins, 2005), which eschews passive‐specific rules or processes altogether (Chomsky, 1993: 4):

Constructions such as…[the] passive remain only as taxonomic artifacts, collections of phenomena explained through the interaction of the principles of UG, with the values of the parameters fixed.

Pinker's semantic constraint approach would seem to predict a gradient, such that verbs that are highly consistent with this semantic characterization will be readily passivizable (1–2), with those that are less consistent resisting passivization to a lesser (3) or greater extent (4–5). A purely syntactic approach would seem to predict no such gradient (though completely unpassivizable verbs, e.g., 4–5, could be flagged as such in the lexicon).

At first glance, the findings of a number of forced‐choice comprehension studies appear to provide support for Pinker's approach (Fox & Grodzinsky, 1998; Gordon & Chafetz, 1990; Hirsch & Wexler, 2006; Horgan, 1978; Maratsos, Fox, Becker, & Chalkley, 1985; Meints, 1999; Sudhalter & Braine, 1985). When presented with a passive sentence (e.g., *Bob was kicked by Wendy*) and asked to select the matching picture (e.g., Wendy kicking Bob or Bob kicking Wendy), children generally show better performance for *agent‐patient* and *theme‐experiencer* verbs (e.g., *kick*;* frighten*) than *experiencer‐theme* verbs (e.g., *hear*).

On closer inspection, however, these comprehension findings constitute little support for Pinker's approach for two reasons. The first is that all of these studies were conducted with children. Thus the findings are consistent with the possibility that, while children may *start out* with a semantic passive construction prototype (perhaps based around a few relatively high‐frequency exemplars), adults have a wholly abstract representation, with semantics playing no role. The second, is that a more recent comprehension study found no support for this semantic constraint, for either children or adults (the two groups did not differ significantly). Messenger, Branigan, McLean, and Sorace (2012) replicated the familiar finding of better performance for passive sentences with *agent‐patient* verbs (*bite, carry, hit, pat, pull, squash*) and *theme‐experiencer* verbs (*annoy, frighten, scare, shock, surprise, upset*) than *experiencer‐theme* verbs (*hear, ignore, like, love, remember, see*), but—crucially—found the same pattern for active control sentences. This suggests that participants do not have difficulty with *experiencer‐theme* PASSIVES, but with *experiencer‐theme* VERBS. Presumably this difficulty arises because *experiencer‐theme* verbs reverse the canonical role assignment exemplified by *agent‐patient* and *theme‐experiencer* verbs (see Hartshorne, Pogue, & Snedeker, 2015; Hartshorne & Snedeker, 2013; Hartshorne et al., submitted), and are also more difficult to illustrate and interpret in a picture‐matching task. Note that none of the previous comprehension studies reviewed above included these crucial active control sentences. Thus, in summary, the findings of previous comprehension studies do not provide support for Pinker's putative semantic constraint on the English passive.

A similar conclusion can be drawn with regard to structural priming studies, the findings of which would seem to be more consistent with approaches that treat the passive as a purely syntactic phenomenon. Many adult studies (see Pickering & Ferreira, 2008; for a review) have shown that hearing a passive sentence increases the likelihood of subsequently producing a passive sentence, regardless of the particular verb used (and regardless of its consistency with Pinker's proposed semantic constraint). This effect holds when the verbs (and other material) of the prime and target sentences are semantically unrelated (Bock, 1986; Estival, 1985), and even when the two sentences are from different languages (Hartsuiker, Pickering, & Veltkamp, 2004). Various follow‐up studies have ruled out non‐syntactic explanations based on the re‐use of lexical material (Saffran & Martin, 1997), priming of syntactic roles (Bock & Loebell, 1990; Potter & Lombardi, 1998), animacy (Bock, Loebell, & Morey, 1992) and prosodic contours (Bock & Loebell, 1990). Similar effects have also been observed for children (Bencini & Valian, 2008; Crain & Fodor, 1993; Huttenlocher, Vasilyeva, & Shimpi, 2004; Messenger, Branigan, & McLean, 2011; Savage, Lieven, Theakston, & Tomasello, 2003, 2006).

Indeed, in addition to the comprehension study discussed above, Messenger et al. (2012) also conducted a syntactic priming study that looked specifically at the issue of by‐verb semantic differences. For neither adults nor children (again, the two groups did not differ significantly) did *agent‐patient* verbs (e.g., *kick*), *theme‐experiencer* verbs (e.g., *frighten*) or *experiencer‐theme* verbs (e.g., *see*) differ in their propensity to prime the production of passive sentences (all with *agent‐patient* verbs), even though these three classes differ along Pinker's affectedness gradient (from greatest to least).

Thus, at present, the available experimental data constitute no evidence for—and perhaps even direct evidence against—Pinker's affectedness constraint on the passive in the adult grammar. We suggest, however, that three features of previous studies may have worked against the possibility of observing such an effect.

The first is that these studies used a relatively coarse measure of verb semantics: a categorical division into *agent‐patient, theme‐experiencer* and *experiencer‐theme* verbs. It may be that this measure is insufficiently fine‐grained to capture the relevant by‐verb semantic difficulties. The present studies address this possibility by using instead composite ratings of 10 semantic properties chosen to capture in detail the nature of the putative semantic constraint.

The second is that these studies used online measures (forced‐choice comprehension and production priming) that monitor the language system as it processes language in real time. Such measures might miss a fine‐grained probabilistic semantic constraint, because minor semantic infelicities are relatively unimportant, provided that they do not interfere with the system's ability to arrive at a parse that is “good enough” (in the sense of Ferreira, Bailey, & Ferraro, 2002). It may be that an offline judgment task is more suited to detecting very subtle instances of infelicity, such as passives that violate a semantic constraint. Indeed, many sentences that are rated as ungrammatical in judgment tasks (e.g., *The key to the cabinets are missing) frequently pass unnoticed in online tasks (Bock & Miller, 1991; Clifton, Frazier, & Deevy, 1999; Pearlmutter, Garnsey, & Bock, 1999; see Lewis & Phillips, 2015).

Further suggestive evidence for this possibility comes from a study of fully grammatical but implausible passives (Ferreira, 2003). On around 25% of trials, participants incorrectly interpreted implausible passives (e.g., *The dog was bitten by the man*) by reversing the roles (e.g., as “The man was bitten by the dog”). Again, the processing mechanism arrives at a plausible interpretation, sometimes bypassing a full parse altogether, and so misses violations (here, of plausibility rather than syntactic or semantic constraints) that are (presumably) readily noticed in an offline judgment task. The present studies address this possibility by using both a time‐sensitive online comprehension measure and an offline graded judgment measure, with the same stimuli.

The use of these dependent measures also addresses a third feature of previous studies that may have worked against the possibility of observing fine‐grained by‐verb semantic differences on passivizability. Both forced‐choice comprehension and production priming yield a binary dependent measure: On each trial, a passive sentence is either comprehended/produced or it is not, there is no half‐way house. Thus even a passive that violates a semantic constraint may be sufficient to tip the scales in favor of the correct picture in a comprehension task, and of the passive construction (as opposed to the active) in a priming task. Indeed, given that even highly ungrammatical sentences (e.g., *Me fell over; *Lisa poured the rug with juice; *£5 was cost by the book) are often readily interpretable, it would be surprising if much more minor infelicities (e.g., *?Bob was seen by Wendy*) interfered with adults' ability to select the matching picture in a comprehension study, or to show syntactic priming in a production study. The present studies address this possibility by using continuous dependent measures: a 5‐point scale in the grammaticality judgment task and a reaction time measure in the forced‐choice comprehension task.

In summary, while previous studies do not provide support for the existence of a semantic affectedness constraint on the English passive, it would seem premature to reject this possibility without first addressing some of the features of previous studies that may count against the possibility of observing such an effect. We begin (Study 1) by obtaining a fine‐grained measure of the proposed semantic constraint: adult ratings of the extent to which a large number of verbs (*N *=* *475, chosen to represent all of the relevant verb types listed by Pinker, 1989; and Levin, 1993), exhibit each of 10 semantic properties pertaining to “affectedness” (Pinker, 1989; Pinker et al., 1987). Following a similar logic to Messenger et al. (2012)—that is, using active sentences as a control—we then investigate whether the resulting composite semantics measure is a better predictor of the relative acceptability of each of these verbs in the passive than the active construction (Study 2). Next, we investigate whether the pattern of findings observed across all 475 verbs (some of which cannot be grammatically passivized at all) holds when looking only at a core set of 72 verbs that are (in a binary sense) all passivizable. Finally, we use the same set of 72 verbs to investigate whether the composite semantics measure is a better predictor of performance with passive than active sentences in a time‐sensitive forced‐choice animated picture‐matching task. Because the aim is to investigate the nature of passive representations in the adult grammar, all studies are conducted with native‐speaking adults (unlike most studies in this domain, which generally focus on children).

## 2 Study 1: Semantic feature ratings

The aim of this study was simply to derive a composite verb‐by‐verb measure of semantic “affectedness” for use in the subsequent studies.

### 2.1 Participants

Participants were 16 native‐speaking adults (university students) who did not take part in any of the other studies. Participants were paid £50 for completing the semantic rating task.

### 2.2 Verbs

We first selected an extended set of 475 verbs (for use in Experiment 2), by consulting lists of passivizable and non‐passivizable verbs given in Pinker et al. (1987: 250–6) and Levin (1993), making sure that we included all the verbs used in the previous studies of Sudhalter and Braine (1985), Maratsos et al. (1985), Gordon and Chafetz (1990) and Messenger et al. (2012). (This latter constraint entailed including particle verbs such as *cheer up*, partly against our better judgment, given that the syntactic status of these verbs is not entirely clear.) The verbs (see Data S1) were chosen to ensure a good spread along the continuum of “completely passivizable” to “completely non‐passivizable” verbs. A subset of 72 verbs—24 *agent‐patient*, 24 *theme‐experiencer* and 24 *experiencer‐theme* (including all the verbs used by Messenger et al., 2012)—were designated the core set, for use in Experiments 3–4:

1. *agent‐patient*: avoid, bite, call, carry, chase, cut, dress, drop, eat, follow, help, hit, hold, hug, kick, kiss, lead, pat, pull, push, shake, squash, teach, wash.
2. *theme‐experiencer*: amaze, amuse, anger, annoy, bother, calm, cheer up, disgust, distract, disturb, entertain, frighten, impress, irritate, please, sadden, scare, shock, startle, surprise, tease, terrify, upset, worry.
3. *experiencer‐theme*: admire, believe, dislike, fear, forget, hate, hear, ignore, know, like, listen to, look at, love, miss, notice, overhear, recognize, remember, see, smell, spot, trust, understand, watch.

These verbs were selected to be passivizable, reversible and relatively easy to illustrate in animations (again, the decision to match the stimuli from previous studies necessitated the inclusion of three particle verbs—*cheer up*,* listen to,* and *look at*—which, ideally, we would have preferred to avoid).

### 2.3 Semantic ratings

Raters were given the following instructions: “On the following sheet is a list of 475 verbs. Each describes an event involving two people (or things, ideas, etc.), denoted by A and B. For example, if the verb is *damage*, the event would be *A damaged B*. Each column lists a statement. Your task is to rate the extent to which each statement is appropriate for each verb, on a scale of 1–9.” The statements rated corresponded to a set of 10 semantic properties listed by Pinker (1989) as characteristic of the passive construction (on the basis of previous work in theoretical linguistics):

(a) A causes (or is responsible for) some effect/change involving B, (b) A enables or allows the change/event, (c) A is doing something to B, (d) A is responsible, (e) A makes physical contact with B, (f) B changes state or circumstances, (g) B is responsible [predicted to have a negative relationship with passivizability], (h) It would be possible for A to deliberately [VERB] B, (i) The event affects B in some way, (j) The action adversely (negatively) affects B.

Note that these raters did not encounter any passive sentences, or any mention of passives, throughout the rating task. Thus it is extremely unlikely that they would have spontaneously adopted a strategy of using passivizability as a criterion for any of these semantic feature ratings.

For each verb, the mean rating across all 10 raters was taken as the score for the relevant semantic feature. Principle Components Analysis (PCA) was used to reduce these 10 semantic predictor variables to a composite measure of (putative) passive‐consistent semantics. PCA works by collapsing across questionnaire statements to which participants showed a similar pattern of responses across items (here, verbs).

### 2.4 Results

The factor loadings are shown in Table 1, 1.

**Table 1:** Original semantic feature measures and derived predictor (*A affects B*)

Original Feature Rated	Semantic Predictor A Affects B
A causes (or is responsible for) some effect/change involving B	0.916
A enables or allows the change/event	0.762
A is doing something to B	0.874
A is responsible	0.635
A makes physical contact with B	0.714
B changes state or circumstances	0.903
B is responsible	−0.320
It would be possible for A to deliberately [VERB] B	0.642
The event affects B in some way	0.893
The action adversely (negatively) affects B	0.720
Eigenvalue	5.74
% variance explained	57.4

All but one of the 10 original semantic features (“B is responsible,” −0.32) had a large positive loading (≥ 0.64) on a single composite predictor, which we named *A affects B*. This composite predictor accounted for 57% of by‐verb variance; that is, for the variance explained by around 6 of the original 10 predictors (Eigenvalue = 5.74). According to Pinker's proposed affectedness constraint, this variable is predicted to have a positive relationship with passivizability in the subsequent studies (recall that “B” denotes the patient, the subject of the passive). Two further components explained a much smaller amount of additional variance (12% and 10% respectively) and so were not retained.

**Figure 1:**
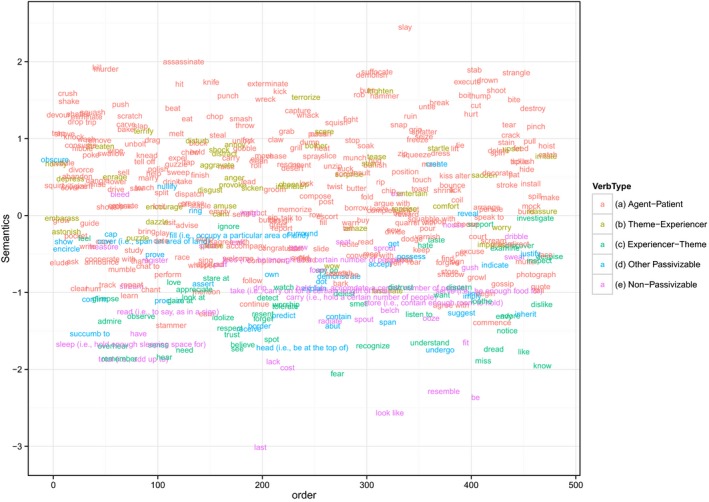
Mean semantic ratings for all 475 verbs. Higher values on the Y axis indicate higher ratings of “affectedness” (i.e., of putative passive‐consistent semantics). The distribution of verbs along the X axis is arbitrary.

Fig. 1 shows the values of each verb on the derived composite semantic predictor (note that the values on the X axis are arbitrary). It is clear that while all of the verbs with very high affectedness scores are *agent‐patient* verbs (and all denote acts of violence; *slay, assassinate, kill, stab, strangle, murder, suffocate*), a number of *theme‐experiencer* verbs (*frighten, terrorize, scare, terrify*) are not far behind, and indeed score higher on this measure than the majority of *agent‐patient* verbs. While this makes intuitive sense—*theme‐experiencer* verbs, by definition, describe an event in which the patient is affected—it suggests that a categorical division between *agent‐patient* and *theme‐experiencer* verbs (e.g., Messenger et al., 2012) is unlikely to be able to capture the types of gradient semantic effects predicted by Pinker's account. In contrast, *experiencer‐theme* verbs (e.g., *fear, hear, see* and *like*) are so non‐affecting that they are intermingled with non‐passivizable verbs such as *cost, sleep, fit* and *total*). Thus to the extent that a categorical division can capture the semantic differences between passivizable verbs (which is not a great extent), the appropriate division is between *agent‐patient* + *theme‐experiencer* verbs on the one hand and *experiencer‐theme* verbs on the other. In particular, note that the distinction between “actional” (i.e., *agent‐patient*) and “psychological” or “mental state” verbs (*theme‐experiencer* + *experiencer‐theme*)—e.g., Maratsos et al. (1985)—is a red herring: *theme‐experiencer* verbs (e.g., *frighten*) are semantically more akin to *agent‐patient* verbs (e.g., kick) than to *experiencer‐theme* verbs (e.g., *hear*).

Having established that, as we speculated in the introduction, a coarse distinction between *agent‐patient*,* theme‐experiencer* and *experiencer‐theme* verbs is not sufficient to characterize Pinker's proposed “affectedness” constraint on the passive, we now proceed to our main question of interest: whether a fine‐grained, continuous measure of affectedness can predict the relative passivizability of verbs in judgment tasks (Studies 2–3) and a comprehension task (Study 4).

## 3 Study 2: Grammaticality judgments (475 verbs)

The aim of this study was to test the prediction that the composite semantic affectedness predictor (see Study 1) will be a better predictor of the by‐verb pattern of acceptability in the passive than the active construction (indicated by an interaction of semantics by sentence type). Recall that we would still expect the measure of affectedness to predict *some* variance in judgments for actives, given that many of the verbs that score low for affectedness (i.e., *experiencer‐theme* verbs such as *hear, ignore, like, love, remember, see*) reverse the canonical role assignment exemplified by *agent‐patient* and *theme‐experiencer* verbs, even in active sentences.

### 3.1 Method

#### 3.1.1 Participants

Participants were 20 adults recruited from the same population as Study 1. None took part in any of the other studies, and each received £20 for their participation.

### 3.2 Materials and procedure

For each of the 475 verbs in the extended set, we created a spreadsheet‐based grammaticality judgment questionnaire containing one active and one passive sentence with the same NPs (e.g., *Homer amused Marge; Marge was amused by Homer*). We then created a second version of the questionnaire by reversing all reversible passives (e.g., *Marge amused Homer; Homer was amused by Marge*) (unlike in the subsequent studies, not all verbs were reversible). We then repeated the entire procedure to create third and fourth versions of the questionnaire with different NPs (e.g., *Bob amused Wendy; Wendy was amused by Bob; Wendy amused Bob; Bob was amused by Wendy*). Participants were randomly allocated to one of the four questionnaires. Within each questionnaire, the order of sentences was randomized on a participant‐by‐participant basis. Participants rated the acceptability of sentences using a 5‐point numerical Likert scale, and were given the following instructions.

In this study, you will rate 950 sentences for grammatical acceptability.

For each sentence, enter a whole number between 1 (completely unacceptable) and 5 (completely acceptable). People tend to differ in their judgments of how acceptable sentences are. Therefore this should not be considered a “test” of your grammar. Acceptability is a sliding scale and not a *yes/no* judgment. It is therefore very important that you try to use the WHOLE of the scale—do NOT just put 1 or 5 for every answer.

Before completing the task, participants completed a training phase consisting of five sentences: one fully acceptable, one fully unacceptable, and three in between (i.e., that typically receive ratings of 2/5, 3/5 and 4/5 in adult studies); see Ambridge, Pine, Rowland, and Young (2008) for details.

#### 3.2.1 Frequency counts

When estimating the influence of a verb's semantic properties on its passivizability, it is important to control for overall verb frequency, on the assumption that participants will show better general task performance for more frequent verbs. It is also important to control for verb frequency *in the passive construction*. Otherwise, we have no way of knowing whether participants show better processing and/or greater acceptance of passive uses of a particular verb because (a) it is consistent with a semantic constraint on the passive or (b) that particular verb simply happens to have occurred frequently in the passive for unrelated reasons (e.g., a pragmatic bias makes passives particularly frequent for verbs such as *sting, bite* and *run‐over*, where humans tend to be the patient, but discourse‐focal). Of course, if there are indeed by‐verb semantic differences in passivizability, one would expect these differences to be reflected to some degree in passive frequency counts (i.e., that semantically passivizable verbs will appear in passive constructions more often). Thus, when looking for by‐verb semantic differences in passivizability, controlling for frequency in the passive constitutes a particularly stringent and conservative control.

Counts of overall and passive frequency were obtained from the British National Corpus (BNC). Passive counts were obtained using a computer program (written by the final author) that searched the corpus for candidate passive sentences. For each verb, the second author hand‐coded 20 candidate passives in order to obtain a by‐verb hit rate that was prorated to yield the final passive count for that verb. All counts were log (*n *+* *1) transformed. Although the BNC includes both written (80%) and spoken texts, this does not constitute a problem, on the assumption that the grammar of literate adult speakers is affected by language encountered in either form.

### 3.3 Results and discussion (Experiment 2)

Many grammaticality judgment studies use difference‐score data, which, in this case, would be calculated by subtracting the rating for each passive sentence from the rating for its active equivalent (e.g., Pinker et al., 1987). However, such a measure would not be appropriate in the present study, given that our goal is to investigate whether the semantic predictor has differential effects on ratings of active and passive sentences. We therefore analyzed the raw ratings for active and passive sentences together including sentence type and its interactions as a factor.

**Figure 2:**
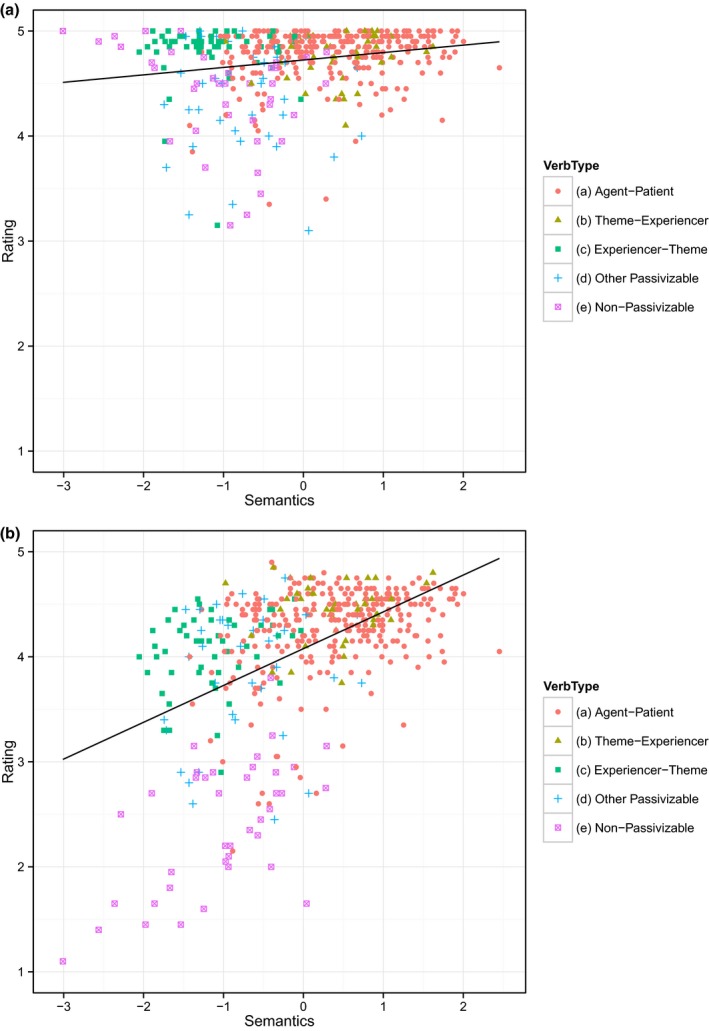
Mean grammaticality judgment score for (a) actives and (b) passives as a function of the semantic predictor (475 verbs; Study 2).

All analyses—for this and subsequent studies—consisted of linear mixed effects regression models, calculated using the *lmer* function of the *lme4* package in R (R Core Team, 2012). Participant and verb were included as random effects. Each model included as many by‐participant random slopes as possible without causing convergence failure (by‐verb random slopes are not meaningful given the design). All models included the following predictor variables, which were standardized using a *z*‐score transformation: (a) Overall verb frequency, (b) Verb frequency in the passive construction, (c) Semantic feature measure: *A affects B*. In accordance with the recommendations of a recent paper (Wurm & Fisicaro, 2014), we used simultaneous regression models with no residualization. *P* values were obtained using the backwards model‐comparison procedure, performed automatically using the step feature from the lmerTest package (Kuznetsova, Brockhoff & Christensen, 2013). The analysis is summarized in Table 2. Figs. 2a and 2b plot acceptability judgments on the 5‐point scale, for actives and passives respectively, as a function of the semantic predictor (*A affects B*).

**Table 2:** Experiment 1: Grammaticality judgments for 475 verbs in active and passive sentences

	*B*	*SE*	*t*	*Sum Sq*	*Mean Sq*	*F*	*p*
(Intercept)	4.73	0.08	58.09				
Sentence type (P vs. A)	−0.65	0.01	−60.65	1,981.61	1,981.61	3,678.94	.000***
Total verb freq	0.06	0.02	2.63	0.85	0.85	7.10	.008**
Passive verb freq	0.03	0.02	1.11	21.06	21.06	45.89	.000***
Semantics	0.08	0.02	3.65	41.95	41.95	77.14	.000***
Stype × Total verb freq	−0.24	0.01	−17.30	67.33	67.33	299.23	.000***
Stype × Pass verb freq	0.27	0.01	19.68	303.57	303.57	387.33	.000***
Stype × Semantics	0.22	0.01	19.10	198.29	198.29	364.65	.000***

*Note*. *p < 0.05, **p < 0.01, ***p < 0.001.

All main effects were significant, indicating that grammatical acceptability increases with sentence type (passive < active), total verb frequency, passive verb frequency and semantic affectedness. The interactions indicate that (a) total verb frequency has a greater effect for actives than passives, while both (b) passive verb frequency and—crucially— (c) semantic affectedness have a greater effect for passives than actives (see Fig. 2).

Thus while there is some evidence to suggest a general dispreference for verbs that reverse canonical marking, even for active sentences (e.g., Messenger et al., 2012), the finding of a significant interaction, such that the by‐verb effect of passive‐consistent semantics is greater for passive than active sentences, constitutes support for Pinker's proposed semantic constraint on the passive.

## 4 Study 3: Grammaticality judgments (core set of 72 passivizable verbs)

A possible objection to the conclusion above is that the semantic effect observed could be driven mainly or entirely by the non‐passivizable verbs (e.g., *cost, weigh*) which constitute clear outliers (see Fig. 2). On this interpretation, all that our “semantic” predictor is doing is picking out verbs that are non‐passivizable, perhaps even for syntactic reasons. For example, Newmeyer (2015: 22) argues that “Passives [such as] *A lot of money was cost by the book and *180 pounds was weighed by John are impossible because the post‐verbal phrases are not arguments of the verb. The “prototypicality” of the verb does not enter directly into the explanation” (scare quotes in original). One way to rule out this objection would be to show that the verb semantics measure predicts the relative acceptability of passives—to a greater extent than actives—even when looking across a set of passivizable verbs.

### 4.1 Method

#### 4.1.1 Participants

Participants were 16 adults recruited from the same population as Studies 1 and 2. None took part in any of the other studies, and each received either course credit or £10 for their participation.

#### 4.1.2. Stimuli

This study used the core set of 72 verbs (see Study 1): 24 *agent‐patient* verbs (e.g., *bite, carry, hit, pat, pull, squash*), 24 *theme‐experiencer* verbs (e.g., *annoy, frighten, scare, shock, surprise, upset*) and 24 *experiencer‐theme* verbs (e.g., *hear, ignore, like, love, remember, see*). Importantly, all of these verbs are passivizable in a binary sense, with even the least acceptable passive (with *believe*) receiving a mean rating of 3.5/5. Syntactically, all verbs clearly select two argument NPs (c.f., Newmeyer, 2015). For each verb, we created two active and two passive sentences with the same NPs (e.g., *Homer amused Marge, Marge was amused by Homer*;* Marge amused Homer, Homer was amused by Marge*) and suitable animations (e.g., Homer causing Marge to laugh and vice versa). Participants rated a single active‐passive sentence pair, matched for participant roles, for all 72 verbs (e.g., half rated *Homer amused Marge* and *Marge was amused by Homer*; half rated *Marge amused Homer* and *Homer was amused by Marge*), for a total of 144 trials per participant. The full set of verbs (S1) and animations (S2) can be found in the Supplementary Material available online.

#### 4.1.2 Procedure

The sentences and accompanying animations were presented in random order (different for each participant), using iTunes (www.apple.com/itunes). Participants supplied their ratings using a 5‐point “smiley‐face” scale, originally designed for use with children (see Ambridge et al., 2008, for details). The scale consists of a color‐coded horizontal array of five faces, ranging from saddest (ungrammatical) to happiest. The two saddest faces are red, the two happiest green, and the middle face half red, half green. Participants marked their answers in a booklet containing 144 copies of the scale. The same practice trials as for Study 2 were used to demonstrate the use of the scale.

### 4.2 Results

**Figure 3:**
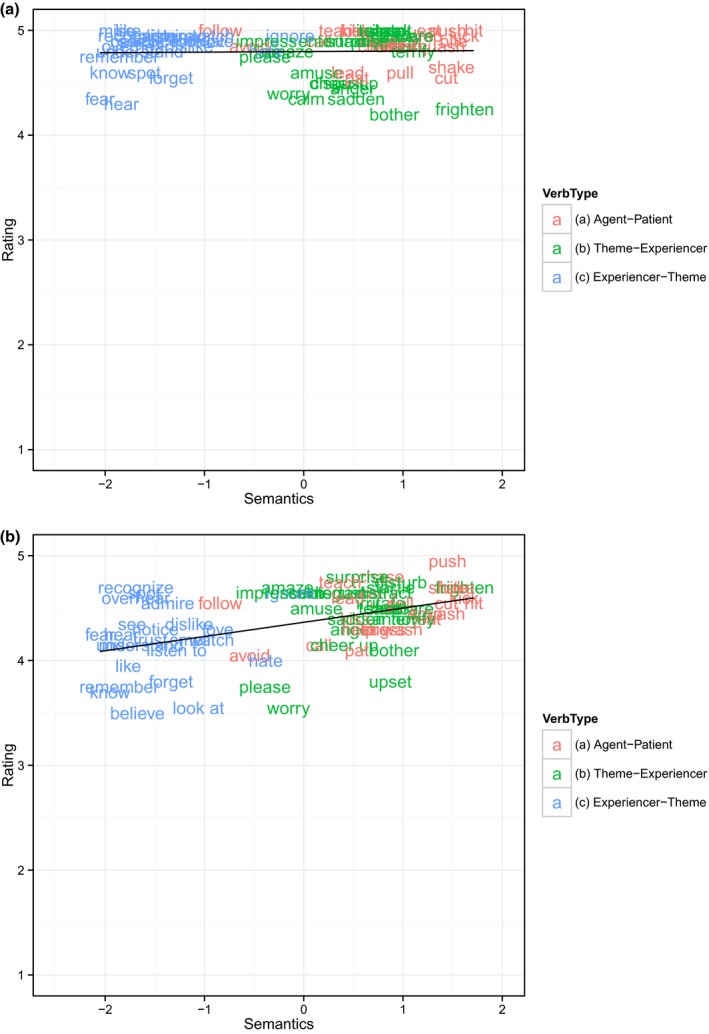
Mean grammaticality judgment score for (a) actives and (b) passives as a function of the semantic predictor (72 passivizable verbs; Study 3).

The data were analyzed in the same way as for Study 2, and showed exactly the same pattern (see Table 3 and Fig. 3).

**Table 3:** Experiment 3 grammaticality judgments for 72 verbs in active and passive sentences

Judgments: Core Set (72 Verbs)							
	*B*	*SE*	*t*	*Sum Sq*	*Mean Sq*	*F*	*p*
(Intercept)	4.78	0.07	71.46				
Sentence type (P vs A)	−0.41	0.02	−17.53	135.63	135.63	307.28	.000***
Total verb freq	0.03	0.03	0.78	0.80	0.80	6.87	.011*
Passive verb freq	0.03	0.03	0.79	5.86	5.86	14.70	.000***
Semantics	0.01	0.03	0.55	1.96	1.96	5.68	.021*
Stype × Total verb freq	−0.21	0.03	−6.70	14.31	14.31	44.86	.000***
Stype × Pass verb freq	0.16	0.03	5.62	13.28	13.28	31.59	.000***
Stype × semantics	0.08	0.02	4.05	5.67	5.67	16.39	.000***

*Note*. *p < 0.05, **p < 0.01, ***p < 0.001.

All main effects were significant, indicating that grammatical acceptability increases with sentence type (passive < active), total verb frequency, passive verb frequency and semantic affectedness. The interactions indicate that (a) total verb frequency has a greater effect for actives than passives, while both (b) passive verb frequency and—crucially— (c) semantic affectedness have a greater effect for passives than actives (see Fig. 3).

Thus, exactly as for Study 2, the finding of a significant interaction, such that the by‐verb effect of passive‐consistent semantics is greater for passive than active sentences, constitutes support for Pinker's proposed semantic constraint on the passive. Crucially, since all verbs were passivizable, and all NPs were syntactic arguments of the verb, this finding cannot be due to a confound introduced by the use of non‐passivizable verbs for which the (would be) passive subject is not an argument of the verb (Newmeyer, 2015).

## 5 Study 4: Forced‐choice comprehension with RT measure (core set of 72 passivizable verbs)

The findings of Studies 2–3 constitute support for the claim of a probabilistic semantic constraint on the passive construction in the adult grammar. This raises the issue of why the forced‐choice comprehension study of Messenger et al. (2012) failed to find such an effect for adults (or children). Are “fast” online measures inherently unsuited to detecting such subtle effects, or could other features of the design of this previous study—in particular the categorical nature of both the semantic predictor variable and the binary (correct/incorrect) outcome variable—be responsible?

In order to investigate this issue, we conducted a timed forced‐choice animated picture‐matching study. On each trial, the participant heard a sentence (e.g., *Marge was amused by Homer*) and was asked to indicate, as quickly as possible, whether the description matched the animation on the left‐ or right‐hand side of the screen (e.g., Marge amusing Homer / Homer amusing Marge), by pressing one of two computer keys aligned with the left‐ and right‐hand sides of the screen, respectively.

### 5.1 Method

#### 5.1.1 Participants

Participants were 16 adults recruited from the same population as Studies 1–3. None took part in any of the other studies, and each received either course credit or £10 for their participation.

#### 5.1.2 Stimuli

The sentences and animations were the same as those used in Study 3 (based on the core set of 72 passivizable verbs). Participants completed one active and one passive trial for each of 36 verbs (half of the total), for a total of 72 trials per participant. Testing was carried out over 2 days. For half of the verbs, participants heard the passive sentence on Day 1 and the active sentence on Day 2, with this pattern reversed for the other half (the two batches were created at random on a participant‐by‐participant basis). Trials were counterbalanced for (a) whether the target was on the left‐ or right‐hand side for the active trial, (b) whether the target for the passive trial was on the same or opposite side to the target for the active trial with the same verb and (c) whether the participant roles were the same for the active and passive sentence for each verb (e.g., *Homer amused Marge / Marge was amused by Homer*) or different (e.g., *Homer amused Marge / Homer was amused by Marge*). The direction in which the action unfolded was not counterbalanced but standardized: right‐to‐left in the left‐hand video and left‐to‐right in the right‐hand video. In order to further aid disambiguation, the left‐ and right‐hand videos had beige and white backgrounds, respectively, and the left‐hand video was slightly higher on the screen.

#### 5.1.3 Procedure

Trials were presented in random order (different for each participant) using the software package *Processing*. The procedure for each trial was as follows. First the participant placed one finger of each hand on the response keys. Next, the animations were previewed; first the left‐hand animation, accompanied by the audio “Look at these two. I wonder what's happening here,” then the right‐hand animation, accompanied by the audio “Oh look! Now it's the other way around.” Then both animations played together, with the movements of the two agents and two patients synchronized.

When the animations ended, freeze‐frames of the end‐point of the animations remained on screen. The animations were designed such that these freeze‐frames alone, in principle, provided sufficient information to allow participants to choose the correct referent for the audio, even without the animation. For example, where the motion of one character is key (e.g., *Marge avoided Homer*) an arrow indicating the direction of the now‐completed motion remained on screen. The intention was to ensure that the task had all the advantages of a standard still‐picture‐matching task, while using animations to provide additional information.

Once the freeze frame picture was on screen, sentence playback began. The audio recordings were standardized so that the disambiguation point (the onset of the main verb) always occurred exactly 7 s after the start of the target animations. At the disambiguation point, the timer started and ran until the participant pressed either the left‐ or right‐hand key. When the key was pressed, a cartoon hand appeared on screen to indicate the participant's choice (any further presses were not recorded). The screen then went blank, ready for the participant to initiate the next trial.

### 5.2 Results

**Figure 4:**
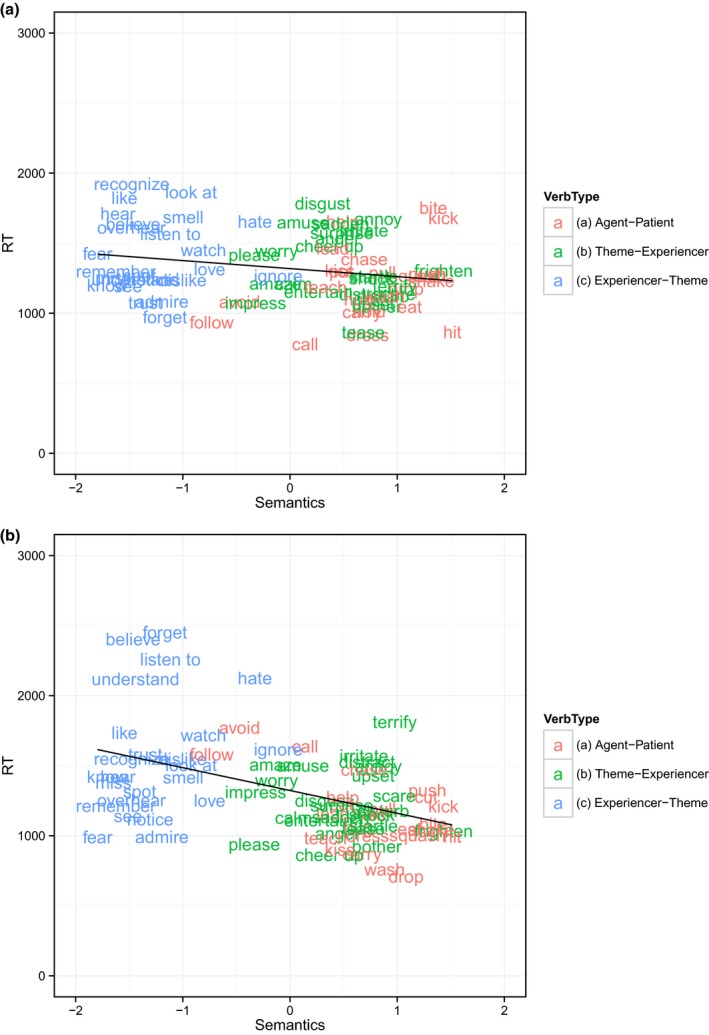
Mean reaction time for (a) actives and (b) passives as a function of the semantic predictor (72 passivizable verbs; Study 3).

The dependent measure was participants' reaction time, excluding any trials with RTs > 10 s and/or incorrect responses (although, in practice, the majority of participants performed at ceiling for both actives and passives). Because mean RTs for the passives and actives might be expected to differ, with longer RTs for passives, we standardized the RTs into *z*‐scores for passives and actives separately. This ensures that any larger effect for passives than actives is not a simple consequence of the fact that it is easier to take a fixed amount (e.g., 500 ms) off a longer than shorter reaction time. The data were analyzed in the same way as for Studies 2–3 (see Table 4 and Fig. 4).

**Table 4:** RT for correct picture‐choices for 72 verbs in active and passive sentences

	*B*	*SE*	*t*	*Sum Sq*	*Mean Sq*	*F*	*p*	*sig*
(Intercept)	−0.09	0.17	−0.56					
Sentence type (P vs A)	0.01	0.04	0.20	0.01	0.01	0.04	.839	n.s
Total verb freq	−0.08	0.04	−2.19	0.03	0.03	1.50	.228	n.s
Semantics	−0.11	0.04	−2.67	10.49	10.49	22.00	.000	***
Stype × Total verb freq	0.09	0.04	2.06	4.53	4.53	4.25	.040	*
Stype × Semantics	−0.10	0.04	−2.25	2.45	2.45	5.04	.025	*
Eliminated								
Stype × Pass verb freq	NA			0.00	0.00	*0.08*	*.775*	n.s
Passive verb freq	NA			1.31	1.31	2.54	.117	n.s

*Notes*. n.s = not significant.

*p < 0.05, **p < 0.01, ***p < 0.001.

Focusing first on the control predictors, both the main effect of passive verb frequency and its interaction with sentence type were non‐significant, and so were eliminated by the backwards model‐comparison procedure. The main effects of sentence type and total verb frequency were also non‐significant, but were not eliminated, due to their involvement in significant interactions. The significant interaction of sentence type by total verb frequency indicates that verb frequency has a greater effect on speeding up reaction times for active than passive sentences.

Turning now to the findings of interest, the main effect of semantics was significant, indicating that—for active and passive sentences alike—reaction time decreases as semantic affectedness increases. Thus again, the findings are indicative of a general dispreference for verbs that reverse canonical marking (i.e., *experiencer‐theme* verbs), even for active sentences (e.g., Messenger et al., 2012; Hartshorne & Snedeker, 2013; Hartshorne et al., in press; Hartshorne, O'Donnell, Sudo, Uruwashi & Snedeker, submitted). Crucially, however, the significant interaction of the semantic predictor by sentence type indicates that affectedness has a greater effect on speeding up reaction times for passive than active sentences.

Thus, exactly as for Studies 2–3, the finding of a significant interaction, such that the by‐verb effect of passive‐consistent semantics is greater for passive than active sentences (even when all passives are grammatical), constitutes support for Pinker's proposed semantic constraint on the passive. This finding suggests that the previous null finding of Messenger et al. (2012) could be due to the use of an insufficiently sensitive semantic measure (i.e., verb class) and/or dependent measure (i.e., correct/incorrect picture choice).

## 6 General discussion

The aim of the present study was to investigate the psychological reality of a semantic constraint on the passive in the adult grammar, originally proposed by Pinker et al. (1987; see also Pinker, 1989). This “affectedness” constraint was posited to explain the phenomenon that certain verbs appear to resist passivization altogether (e.g., *£5 was cost by the book). However, the findings of several previous comprehension and production priming studies cast doubt on the existence of this constraint: Passives with *experiencer‐theme* verbs (e.g., *see*), which score low for affectedness, did not differ from either *agent‐patient* (e.g., *kick*) or *theme‐experiencer* verbs (e.g., *frighten*) in their propensity to prime the production of passive sentences, and—relative to actives—did not show any particular decrement in picture‐choice tasks.

The present study investigated whether these null effects could result in part from the paradigms used in these previous studies. These involved (a) a categorical measure of verb semantics (*agent‐patient* / *theme‐experiencer* / *experiencer‐theme* verbs), (b) a “fast” online task (comprehension or production priming) and (c) a binary outcome variable (passive comprehended/produced or not). In contrast, the present study used (a) a graded measure of verb semantics (semantic ratings from adult native speakers), (b) a “slow” offline judgment task, as well as a “fast” online comprehension task and (c) graded outcome variables (grammatical acceptability on a 5‐point scale / comprehension reaction time).

With these modifications in place, all three studies—(a) grammaticality judgments with 475 verbs, (b) grammaticality judgments with 72 passivizable verbs and (c) forced‐choice animated picture matching with the same 72 verbs—found evidence for Pinker's proposed semantic constraint on the passive. Although, in each study, the semantic measure predicted performance across both actives and passives (presumably because *experiencer‐theme* verbs, which reverse canonical marking, are just difficult in general), a significant interaction was observed, such that the facilitatory effect of passive‐consistent semantics (“affectedness”) was greater for passive than active sentences.

The conclusion is that Pinker's semantic constraint on the passive (or something very like it) is psychologically real, and must therefore be incorporated into any account of the underlying adult grammar. This does not necessarily require us to adopt any one theoretical standpoint with regard to the nature of this grammar. For example, the semantic constraint could be implemented as a graded constraint on a lexical non‐movement rule relating actives and passives, as in frameworks such as Lexical Functional Grammar or Head Driven Phrase Structure Grammar (Bresnan, 2001; Pinker, 1989; Pollard & Sag, 1994).

Alternatively, under construction grammar approaches (e.g., Croft, 2001; Goldberg, 1995; Goldberg & Bencini, 2005), the semantic constraint could be implemented at the construction level, whereby the passive construction itself (or its verb slot) has the relevant semantic properties, which the learner acquires by abstracting across concrete utterances that instantiate these properties. An advantage of this approach is that it brings the passive into line with the findings of our recent research on other constructions such as the locative (Ambridge, Pine, & Rowland, 2012), dative (Ambridge, Pine, Rowland, Freudenthal, & Chang, 2014) and reversative *un‐* prefixation (Ambridge, 2013; Blything, Ambridge, & Lieven, 2014). Indeed, while maintaining that “lexical rules are needed, and…meaning‐to‐construction mappings are not enough,” Pinker (2013: xv) himself notes that

the analyses in *Learnability and Cognition* (Pinker, 1989)…are upward compatible with [both] current versions of…Lexical Functional Grammar…and the various versions of Construction Grammar, such as those developed by Ronald Langacker, Adele Goldberg and William Croft. Indeed, my notion of the “thematic core” of an argument structure, which delineates the “conflation class” of verbs compatible with that argument structure [apparently including the passive—BA], is very close to the idea of a “construction meaning” invoked by theories of construction grammar.

Thus the idea of a semantic constraint on the passive—one that is strongly supported by the findings of the present study—is compatible with a variety of different approaches to the adult grammar. One possible exception is more traditional approaches such as Chomskyan X‐bar theory and its descendents (e.g., Minimalism; Chomsky, 1995). The existence of a semantic constraint on the passive is not necessarily incompatible with these approaches. However, the challenge would be to find a way of incorporating some index of relative passivizability in the verb's lexical entry, given that the framework eschews both constructions in general (which Chomsky, 1989:43; dismisses as “taxonomic epiphenomena”), and any passive‐specific construction, rule or process in particular (see quotation from Chomsky, 1993:4; in the Introduction). One possibility might be to posit that underlying passivizability (in a binary sense) is a core grammatical feature listed in a verb's lexical entry, but that the ease of applying this procedure in real time depends on the extent to which the verb's semantics are typical of those that are often used in the passive (or, as Newmeyer, 2003, puts it “grammar is grammar, and usage is usage”). The counterargument is that if one can derive the present results from a unitary process—i.e., compatibility with a semantic construction prototype—it seems unparsimonious to posit a two‐level (i.e., grammar + usage) account.

An assumption that has been implicit thus far is that what determines a verb's passivizability is its lexical meaning (in a kind of fixed dictionary‐definition sense). An alternative possibility that is also consistent with the present findings is that the acceptability of a passive is instead determined by the semantics of the *event*: a passive is grammatical to the extent that the event is construed as one in which the surface subject is affected (indeed, the instructions given were deliberately ambiguous as to whether participants were rating the semantics of the verb *per se*, or the types of event typically denoted by that verb). This alternative is appealing, as it captures the intuition that the grammaticality of a sentence such as ?*Homer was seen by Marge* is much improved in a context in which Homer is *affected* by being seen (e.g., Homer was intending to go to the pub instead of his daughter's recital, but had his plans ruined when he was ***seen by Marge*** en route to the pub). Future studies could test this possibility by having participants rate sentences such as *Homer was seen by Marge* in an “affected” context (as in the example above) and a “neutral” but otherwise similar context (e.g., Homer had agreed to meet Marge in the pub, and so was not particularly affected by her seeing him there when she came to join him).

In the meantime, suggestive evidence comes from the corpus study of Grafmiller (2013). Focusing on *theme‐experiencer* verbs, Grafmiller (2013:202) showed that the probability of a verb occurring in a passive versus an active is correlated with the extent to which the causer is usually something “about which people tend to direct longer lasting attitudes or evaluations”. For example, the verbs for which passives outnumber actives (*fascinate, captivate, concern, horrify, astonish, upset* and *amaze*) are those for which (usually non‐animate) *theme* causes a semi‐permanent state‐change in the (usually animate) *experiencer*. While the present study attempted to control out such factors (i.e., by using relatively neutral contexts and two human NPs wherever possible), we agree that the construal of the event—affected, amongst other things, by the nature of the NPs—is likely to affect the relative acceptability of a passive sentence. Future research is necessary to clarify this issue.

Given that the present study focused on adults, future research will also be needed to mediate between different accounts of the acquisition of the passive by young children (e.g., Borer & Wexler, 1987, 1992; Crain & Fodor, 1993; Brooks & Tomasello, 1999; Israel, Johnson, & Brooks, 2000; Savage et al., 2003, 2006; Huttenlocher et al., 2004; Abbot‐Smith & Behrens, 2006; Bencini & Valian, 2008; Messenger et al., 2011, 2012; Dittmar, Abbot‐Smith, Lieven, & Tomasello, 2013). Particularly relevant is the possibility that children start out with a passive construction that is lexically restricted to prototypical *agent‐patient* verbs (e.g., *kick*), and that gradually broadens to additionally encompass, first, *theme‐experiencer* verbs (e.g., *frighten*) and, later, *experiencer‐theme verbs* (e.g., *see, hear*). A related claim that has been made by authors from otherwise‐opposing theoretical perspectives (e.g., Borer & Wexler, 1987; Israel et al., 2000), is that children start out with adjectival short passives that denote affected states (e.g., *It's wet/got wet; She's scared/got scared*) and only later—as a result of either A‐chain maturation or gradual abstraction respectively—acquire the ability to produce full passives.

Given that we studied only adults, the present findings do not address either of these claims directly. They do, however, count against the possibility that any semantic prototype is an early stepping‐stone that is discarded when children's knowledge becomes more abstract. Rather, they suggest that whether or not a semantic constraint on the passive is operational for young children (and we agree with Messenger et al., 2012, that there is currently no convincing evidence that it is), such a constraint is operational for adults. In future work, we plan to investigate the possibility of an early semantic constraint by adapting the present paradigms for use with young children, and adopting others, such as production priming.

In conclusion, the findings of the present study suggest that passive syntax is indeed semantically constrained in adults. Across three studies, an independent measure of the extent to which individual verbs instantiate semantic properties relevant to the constraint (“affectedness”) significantly predicted the relative acceptability of passive sentences to a greater extent than active sentences. This pattern of findings suggests that any successful model of adults' linguistic knowledge, of whatever theoretical persuasion, will have to incorporate—in some form or other—this probabilistic semantic constraint.

## Supporting information


**Data S1.** Extended set of 475 verbs.
**Data S2.** Animations.

## Note

1 A data file containing the raw semantic ratings for each verb is available from the first author's website: www.benambridge.com
