# Supplementary material for: Is Passive Syntax Semantically Constrained? Evidence From Adult Grammaticality Judgment and Comprehension Studies
Source: Cogn Sci. 2015 Nov 26;40(6):1435–59. doi: 10.1111/cogs.12277 (PMC4996337; doi:10.1111/cogs.12277)
Supplement: Supplementary file 1 — Data S1. Extended set of 475 verbs. Data S2. Animations. [file COGS-40-1435-s001.docx]

**Supplementary Materials** for Ambridge, B., Bidgood, A., Pine, J.M., Rowland, C.F., & Freudenthal, D. (in press). Is passive syntax semantically constrained? Evidence from adult grammaticality judgment and comprehension studies*. Cognitive Science.*

**S1: Extended set of 475 verbs**

**Agent-**

**patient**

abandon

accompany

advise

agree with

alter

announce

appluad

argue with

arrange

ask

assassinate

avoid

bake

banish

bark

beat

begin

bend

bite

block

boil

borrow

bounce

break

bring

build

burn

butter

button

buy

call

capture

carry

carve

catch

chant

chase

chat to

chew

chip

choose

chop

claw

clean

clear

coil

commence

complete

compliment

compose

congratulate

consume

continue

converse with

cook

cooperate with

copy

court

cover

crack

crease

criticize

crush

cuddle

cut

dangle

date

decorate

delete

demolish

desert

design

destroy

devour

direct

disagree with

dispatch

divide

divorce

dodge

drag

draw

dress

drink

drive

drop

drown

dump

earn

eat

eliminate

elude

embrace

empty

end

escort

excuse

execute

expel

exterminate

feed

fight

fill

film

find

finish

fold

follow

forgive

freeze

fry

gobble

gossip

grab

greet

grill

grip

groom

grow

growl

guide

guzzle

hammer

heat

help

hide

hit

hoist

hold

hug

hum

hurt

install

instruct

insult

invent

keep

kick

kill

kiss

knead

knife

knit

knock

lace

lead

learn

leave

lick

lift

lock

lower

make

marry

mash

meet

melt

memorize

mention

mock

move

mumble

munch

murder

mutter

nibble

paint

pat

perform

photograph

pinch

play

pocket

poke

polish

pop

position

post

pot

pour

praise

propose

pull

punch

punish

pursue

push

quarrel with

quote

race

raise

read

recite

record

remove

repeat

report

rescue

reward

rinse

rip

roar

rob

roll

row

ruin

saw

scold

scoop

scratch

scream

seal

search

seize

sell

send

sew

shadow

shake

shatter

shoot

shout

shove

shovel

shrink

sign

sing

sip

sketch

slap

slay

slice

slide

slurp

smash

snap

soak

speak to

spill

spin

splash

splatter

split

spray

squabble with

squash

squeeze

squirt

squish

stab

stain

stammer

staple

start

steal

stop

store

strangle

stroke

study

suck

suffocate

swallow

sweep

tail

take

talk to

tap

teach

tear

tell

tell off

thank

throw

thump

tickle

tie

toast

touch

track

trail

trap

trip

twist

type

unbolt

unlock

untie

unzip

varnish

visit

warn

wash

welcome

whack

whisper

wipe

wreck

write

yell

zip

**Theme-**

**experiencer**

aggravate

amaze

amuse

anger

annoy

astonish

bother

calm

cheer up

comfort

dazzle

depress

disgust

distract

disturb

embarass

encourage

enrage

entertain

fascinate

frighten

horrify

impress

infuriate

irritate

please

provoke

puzzle

reassure

repulse

sadden

scare

shock

sicken

startle

stun

surprise

tease

terrify

terrorize

threaten

upset

worry

wow

**Experiencer-**

**Theme**

admire

adore

appreciate

believe

despise

detect

detest

discern

discover

dislike

distrust

dread

envy

examine

fear

feel

forget

gaze at

glimpse

hate

hear

idolize

ignore

inspect

investigate

know

like

listen to

loathe

look at

love

miss

need

notice

observe

overhear

recognize

remember

resent

respect

see

sense

smell

spot

spy on

stare at

support

taste

tolerate

trust

understand

want

watch

worship

**Other-**

**passivizable**

abut

accept

assert

border

cap

confirm

contain

cover (span area)

create

demonstrate

dot

encircle

explain

fill (occupy area)

get

head (be top of)

imply

indicate

inherit

justify

line

nullify

obscure

own

possess

predict

proclaim

prove

receive

reveal

ring

show

span

succumb to

suggest

surround

undergo

**Non-**

**passivizable**

be

belch

bleed

carry (hold)

cost

dribble

drip

feed (suffice for)

fit

foam

gush

have

hold (accommodate)

house

lack

last

leak

look like

measure

ooze

puff

radiate

read to

register

resemble

seat

serve

sleep (contain)

spew

spout

sprout

spurt

store (suffice to hold)

stream

sweat

take (time)

total (sum to)

use

weigh

**S2: Animations**

(a) Agent-patient verbs

***Avoid*** [A] begins walking directly towards [B] then walks around him. [A] stops at the other side of [B]. An arrow appears indicating [A]’s route. [B] frowns.

***Bite*** [B] is holding out a donut towards [A]. [A] walks towards [B] and leans forward. [A] opens her mouth and eats the donut in one go. Bite marks appear on [B]’s hand. [A] stands straight again. Both [A] and [B] frown.

***Call*** [B] is facing away from [A]. [A] walks towards [B] then stops and raises a hand to his mouth. A speech bubble appears from [A]’s mouth. [B] turns to face [A] and smiles.

***Carry*** [B] is lying in the floor. [A] approaches [B] and picks him up. [A] carries [B] across the screen. [B] smiles.

***Chase*** [A] is standing at the top left of the screen and [B] in the middle. [A] approaches [B]. [B] starts running towards the bottom right and [A] runs after him. They both stop at the bottom right of the screen. [B] smiles. An arrow appears between [A] and [B] indicating the direction they were going.

***Cut*** [B] is standing by a table with a carrot on it. [B]’s hand is directly next to the carrot. [A] approaches the table carrying a knife. [A] cuts the carrot into 3 but also cut’s [B]’s hand. A red line appears on [B]’s hand. Both [A] and [B] frown.

***Dress*** [B] is not wearing a shirt. [A] is holding [B]’s shirt. [A] walks towards [B] holding the shirt then throws it towards him. The shirt goes onto [B]. [B] smiles.

***Drop*** [B] is lying in the floor. [A] approaches [B] and picks him up. [B] stays in [A]’s arms for a second before falling back to the floor. [A] raises her hands to her mouth, which opens. [B] frowns.

***Eat*** A miniature version of [B] stands at the edge of a table. [A] approaches [B] and picks her up. [A] puts opens his mouth wide and puts [B] into it. [A]’s mouth closes with [A]’s legs sticking out. [A]’s legs wiggle.

***Follow*** [A] is standing at the top left of the screen and [B] in the middle. [A] approaches [B]. [B] starts walking towards the bottom right and [A] follows. They both stop at the bottom right of the screen. [B] smiles. An arrow appears between [A] and [B] indicating the direction they were going.

***Help*** [B] is lying on the floor, frowning. [A] approaches [B] then bends down and pulls her to her feet. [B] smiles.

***Hit*** [A] is holding a tennis racket. [A] walks diagonally in front of [B]. [A] swings the racket and hits [B] with it. Both frown.

***Hold*** [A] walks towards [B] and puts her arms around him. They stay in this position. [B] smiles.

***Hug*** [A] walks towards [B] and puts his arms around her. They stay in this position. [B] smiles.

***Kiss*** [A] approaches [B] and leans forward. [A]’s lips extend slightly. [B] does not move except to smile.

***Kick*** [A] and [B] are on opposite sides of the screen. There is a football by [B]’s feet. [A] approaches [B]. [A] kicks the football, which rolls off-screen. As [A] kicks the ball, his foot continues and he also kicks [A]’s leg. [B]’s foot stays in position for half a second to emphasize this. [B]’s foot returns halfway to its original position. Both [A] and [B] frown.

***Lead*** [A] and [B] are on opposite sides at the top of the screen. [A] approaches [B] and takes her hand. [B] turns and points to the diagonally opposite corner of the screen. Both walk in the direction of [A]’s point, with [A] in front. [A]’s hand remains in the pointing position. [B] smiles.

***Pat*** [B] is frowning. [A] approaches [B]. [A] pats [B]’s shoulder several times. [A]’s hand remains on [B]’s shoulder. [B] smiles.

***Pull*** [B] is on roller skates and is holding out a hand. [A] approaches [B] and takes his hand then turns and walks in the opposite direction, pulling [B] behind her. [B] smiles.

***Push*** [B] is on roller skates and facing away from [A]. [A] approaches [B] and pushes his back. [B] rolls across the screen to the other side. [B] smiles. [A]’s arms remain in an extended position.

***Shake*** [B] is sitting in an armchair with closed eyes. [A] approaches [B] and takes hold of [B]’s shoulders. [A] shakes [B]. [B] wakes up and scowls at [A]. [A]’s hands remain on [B]’s shoulders.

***Squash*** [A] is standing on a step. [B] is standing on the floor, facing away from [A]. [A] walks to the edge of the step so that [B] is directly beneath her. [A] presses on [B]’s shoulders, squashing [B] down towards the floor. [B] frowns.

***Teach*** [B] is sitting at a desk facing a blackboard with Chinese characters written on it. [A] approaches the blackboard. [A] indicated the writing with her hand then turns her head to face [B], whilst keeping her hand in place. [B] smiles.

***Wash*** [B] is covered in splashes of mud. [A] is holding a hose pipe. [A] approaches [B]. A jet of water comes out of the hosepipe, covering most of [B]. The jet of water gets smaller but stays on screen. The mud disappears from [B]. [B] smiles.

(b) Theme-experiencer verbs

***Amaze*** [B] is standing looking bored. [A] approaches [B]. [A] performs a back flip, ending with arms raised. [B]’s eyes and mouth open wide.

***Amuse*** [B] is standing looking bored. [A] approaches [B]. [A] performs a silly dance, ending with arms raised. [B]’s eyes open wide and he smiles.

***Anger*** [B] is holding a donut. [A] approaches [B]. [A] takes the donut from [B]’s hand. [B] scowls.

***Annoy*** [B] is holding a donut. [A] approaches [B]. [A] takes the donut from [B]’s hand. [B] scowls.

***Bother*** [B] is sitting in a chair facing a television. [A] walks up to [B] and taps him several times on the shoulder. [B] scowls.

***Calm*** [B] is frowning. [A] walks up to [B] and tears appear at [B]’s eyes. [A] strokes [B]’s arm twice and [B] stops crying and smiles. [A]’s hand remains on [B]’s arm.

***Cheer up*** [B] is frowning. [A] walks up to [B] and pats [B]’s shoulder a few times. [B] smiles. [A]’s hand remains on [B]’s shoulder.

***Disgust*** [A] is holding three cupcakes. [A] walks up to [B] and eats all three cakes. Chocolate smears appear on [A]’s face. [B] scowls.

***Distract*** [B] is sitting in an armchair reading a book. [A] walks up to [B] and taps her shoulder several times. [B] scowls. [A]’s hand remains on [B]’s shoulder.

***Disturb*** [B] is sitting in an armchair reading a book. [A] walks up to [B] and taps her shoulder several times. [B] scowls. [A]’s hand remains on [B]’s shoulder.

***Entertain*** [B] is standing looking bored. [A] approaches [B]. [A] performs a silly dance, ending with arms raised. [B]’s eyes open wide and he smiles.

***Frighten*** [A] is holding a ghost costume. [B] is facing away from [A]. [A] approaches [B] and puts on the costume. [B] turns around and jumps. [B]’s eyes and mouth open wide.

***Impress*** [B] is standing looking bored. [A] approaches [B]. [A] performs a front flip, ending with arms raised. [B]’s eyes and mouth open wide.

***Irritate*** [B] is smiling. [A] walks towards [B] and does a silly dance. [B] scowls.

***Please*** [A] is carrying a bunch of flowers. [A] walks over to [B] and holds up the flowers. [B] raises her hand and the flowers pass to her. [B] smiles. [A]’s had remains in place.

***Sadden*** [B] is holding a hat. [A] approaches [B]. [A] takes the hat from [B]’s hands. [B] frowns.

***Scare*** [A] is holding a ghost costume. [B] is facing away from [A]. [A] approaches [B] and puts on the costume. [B] turns around and jumps. [B]’s eyes and mouth open wide.

***Shock*** [A] is holding a balloon and a pin. [B] is facing away from [A]. [A] approaches [B] and pops the balloon. Small pieces of the balloon remain with lines indicating the popping. [B] turns around and his eyes and mouth open wide.

***Startle*** [A] is holding a balloon and a pin. [B] is facing away from [A]. [A] approaches [B] and pops the balloon. Small pieces of the balloon remain with lines indicating the popping. [B] turns around and his eyes and mouth open wide.

***Surprise*** [A] is has one hand behind his back. [A] walks over to [B] and reveals a bunch of flowers from behind his back. [B] raises her hand and the flowers pass to her. [B] smiles. [A]’s had remains in place.

***Tease*** [A] walks towards [B]. [A] raises her hand to her face, wiggles her fingers and sticke out her tongue. [B] frowns.

***Terrify*** [A] is holding a monster mask. [B] is facing away from [A]. [A] approaches [B] and puts on the mask. [B] turns around and jumps. [B]’s eyes and mouth open wide.

***Upset*** [B] is holding a hat. [A] approaches [B]. [A] takes the hat from [B]’s hands. [B] frowns.

***Worry*** [A] is has one hand behind her back. [A] walks over to [B] and reveals a bunch of spider from behind her back. [A] lifts it up and down in front of [B]. B frowns and looks worried.

c) Experiencer-theme verbs

***Admire*** [B] is standing on a podium holding a trophy. [A] approaches [B], claps and holds out a hand towards [B]. [B] smiles.

***Believe*** [A] approaches [B]. A speech bubble appears from [B]’s mouth with a picture of a ghost inside it. [A] jumps, raises a hand to her mouth and opens her mouth. [B] smiles.

***Dislike*** [A] is facing away from [B], and walks away. A thought bubble appears containing a picture of [B]’s face. A red X appears over this face and [A] scowls. [B] frowns.

***Fear*** [B] is holding a ghost costume. [A] walks towards [B] and [B] puts on the costume. [A] steps back and her mouth and eyes open. [B] smiles.

***Forget*** [A] is facing away from [B] but has a thought bubble containing a picture of [B]’s face. [A] walks away from [B] and the face in the thought bubble fades and disappears. [B frowns.

***Hate*** [A] is facing away from [B], and walks away. A thought bubble appears containing a picture of [B]’s face. A red X appears over this face and [A] scowls. [B] frowns.

***Hear*** [A] and [B] are facing each other. [B] is talking on a mobile phone. A speech bubble appears from [B]’s mouth. [A] approaches [B] and holds up a hand to [A]’s ear. [B] smiles.

***Ignore*** [A] walks towards [B]. [B] does a silly dance. [A] turns away and scowls. [B] frowns.

***Know*** [B], [C] and [D] are standing in a line. [A] approaches them and points at [B], then waves. [B] waves back and smiles. [B]’s hand returns to its original position; [A]’s hand returns to a pointing position.

***Like*** [A] walks towards [B] and raises a hand to indicate [B]. A thought bubble appears from [A]’s head with a heart inside it. [B] smiles.

***Listen to*** [A] walks towards [B]. A speech bubble appears from [B]’s mouth and his mouth opens and closes. [A] nods. [B] smiles.

***Look at*** [A] is facing away from [B] and walks away before turning to face [B]. [B] does a star jump, leaving his arms raised. [A] points at [B]; [B] smiles.

***Love*** [A] walks towards [B] and raises a hand to indicate [B]. A thought bubble appears from [A]’s head with a heart inside it. [B] smiles.

***Miss*** [A] and [B] are facing each other. [A] turns and walks to the side of the screen; [B] turns and leaves the screen. A thought bubble appears from [A]’s head containing [B]’s face. [B] frowns, as does [A] in the thought bubble.

***Notice*** [A] is standing up reading a book. [A] walks towards [B], stops, looks up at [B] and points. [B] smiles.

***Overhear*** [B] is facing away from [A]. [B] is talking on a mobile phone. A speech bubble appears from [B]’s mouth. [A] approaches [B] and holds up a hand to [A]’s ear. [B] frowns.

***Recognize*** [B], [C] and [D] are standing in a line. [A] approaches them and points at [B], then waves. [B] waves back and smiles. [B]’s hand returns to its original position; [A]’s hand returns to a pointing position.

***Remember*** [A] is facing away from [B]. [A] walks away, but stops when a thought bubble containing [B]’s face appears above his head. [A] turns and walks back to [B]. [B] smiles.

***See*** [A] is facing away from [B] but walks in his direction. [A] looks around before looking directly at [B] and pointing in that direction. [B] smiles.

***Smell*** [A] walks towards [B] and leans forwards slightly. Green lines, indicating an odor, emanate from [B]. [A]’s nose wiggles and he frowns. [B] also frowns.

***Spot*** [A] is facing away from [B] but walks in his direction. [A] looks around before looking directly at [B] and pointing in that direction. [B] smiles.

***Trust*** [B] and [C] are standing next to each other. [A] stands apart, holding a handful of money and facing [B] and [C]. [A] walks towards the others and give the money to [B]. [B] smiles.

***Understand*** [A] is frowning. [A] walks towards [B]. A speech bubble appears from [B]’s mouth and her mouth opens and closes. [A] nods his head twice then smiles. [B] also smiles.

***Watch*** [A] is facing away from [B] and walks away. [A] stops and turns to face [B]. [B] starts doing star jumps and [A] points at [B]. [B] smiles.
